# The Association Between Body Mass Index and Arthritis Among US Adults: CDC’s Surveillance Case Definition

**Published:** 2009-03-15

**Authors:** Diane B. Wilson, Jamie M. Zakkak, Jack O. Lanier

**Affiliations:** Departments of Internal Medicine and Massey Cancer Center, Virginia Commonwealth University; Virginia Commonwealth University School of Medicine, Richmond, Virginia; Virginia Commonwealth University School of Medicine, Richmond, Virginia

## Abstract

**Introduction:**

The Centers for Disease Control and Prevention modified the surveillance case definition of arthritis to a more stringent form in 2002. To date, the association between arthritis and obesity (an established risk factor for arthritis) has not been examined with the new definition. We describe the association between body mass index (BMI) (kg/m^2^) and arthritis using the new arthritis case definition to provide a more accurate assessment of the relationship between weight and arthritis among US adults.

**Methods:**

We used data from the 2005 Behavioral Risk Factor Surveillance System (N = 356,112) and univariate and multivariate analyses to assess the relationship between BMI and arthritis among US adults.

**Results:**

Overall, 26% of US adults had self-reported arthritis. Obese respondents (BMI ≥30.0 kg/m^2^) were 1.9 times more likely to report arthritis compared with normal-weight respondents (BMI <25.0 kg/m^2^), and distinguishing between obese levels revealed an even greater association between BMI and arthritis (class III obesity [BMI ≥40.0], odds ratio [OR] = 3.3, 95% confidence interval [CI] = 3.1-3.6; class II obesity [BMI 35.0-39.9 kg/m^2^], OR = 2.5, 95% CI = 2.3-2.7; class I obesity [BMI 30.0-34.9], OR = 1.9, 95% CI = 1.8-2.0).

**Conclusion:**

BMI is an independent risk factor for self-reported arthritis. Maintaining a healthy weight may delay the onset of arthritis. More research is needed to determine the effect of weight loss on the progression of arthritis in overweight individuals.

## Introduction

The prevalence of both obesity and arthritis in the United States continues to rise, and the medical, physical, and social costs associated with these conditions are enormous (1-7). Previous research has identified an association between obesity, defined by using body mass index (BMI) and certain types of arthritis (8-13), yet population-based studies on weight and arthritis in the United States are limited. Additionally, the Centers for Disease Control and Prevention (CDC) changed the surveillance case definition of arthritis in 2002 to a more exclusive form that only includes self-reported, doctor-diagnosed arthritis (http://www.cdc.gov/arthritis/data_statistics/case_def_additional.htm). To our knowledge, existing studies used the old, more inclusive definition. From 1996 through 2001, the case definition included people with doctor-diagnosed arthritis and/or those with chronic joint symptoms; since 2002, the definition of arthritis is defined as a yes answer to the following question: "Have you ever been told by a doctor or other health professional that you have some form of arthritis, rheumatoid arthritis, gout, lupus, or fibromyalgia?" Given the growing public health importance of obesity and arthritis, we aim to more accurately describe the relationship between BMI and arthritis among US adults.

Our 2 primary objectives are 1) to describe the prevalence of self-reported arthritis among US adults by various demographic and socioeconomic characteristics and 2) to determine whether BMI is significantly associated with arthritis at the population level, using the revised arthritis definition. Study findings will provide a more accurate assessment of the relationship between weight and arthritis in the United States and will enhance related clinical medical interventions and public health initiatives.

## Methods

Data from the 2005 CDC Behavioral Risk Factor Surveillance System (BRFSS), an annual, state-based, random-digit–dialed telephone survey that collects information on health and risk behaviors for noninstitutionalized civilians aged 18 years or older, were used to examine the relationship between arthritis and BMI among adults in all 50 states, the District of Columbia, Puerto Rico, the US Virgin Islands, and Guam. BRFSS data were downloaded from the CDC Web site for this analysis, and all respondents who had answered the question pertaining to arthritis status were included (N = 356,112) (14). Respondents were defined as having arthritis if they answered yes to the question, "Have you ever been told by a doctor or other health professional that you have some form of arthritis, rheumatoid arthritis, gout, lupus, or fibromyalgia?" This method of identifying people with arthritis is recommended by CDC (15) and has moderate sensitivity (ability to correctly identify people with arthritis) (70.3%) and specificity (ability to correctly identify people without arthritis) (72.4%) (16). BMI (kg/m^2^) was calculated from self-reported height and weight and was classified into 5 categories: normal (<25.0), overweight (25.0-29.9), class I obesity (30.0-34.9), class II obesity (35.0-39.9), and class III obesity (≥40.0).

All other variables we examined were categorical: sex, race/ethnicity (non-Hispanic white, non-Hispanic black; Hispanic; other), age (18-34, 35-44, 45-54, 55-64, ≥65 years), education (less than high school graduate, high school graduate, attended college, college graduate), annual income (<$25,000, $25,000-$49,999, ≥$50,000), health insurance coverage (insured or uninsured), and physical activity (PA) level (active, insufficient activity, and inactive). PA categories were based on the Surgeon General's recommendations (17). "Active" was defined as meeting the recommendations (30 minutes or more of moderate-intensity PA on 5 or more days per week or 20 minutes or more of vigorous-intensity PA on 3 or more days per week) during the preceding month, "insufficient activity" was defined as not meeting the recommendations during the preceding month, and "inactive" was defined as reporting no PA outside of one's occupation during the preceding month.

SAS version 9.1 (SAS Institute Inc, Cary, North Carolina) was used for all analyses. Descriptive statistics were calculated to describe the population, and the prevalence of arthritis across population groups was reported with corresponding 95% confidence intervals (CIs). Univariate analyses were conducted to obtain the crude odds ratios (ORs) and to estimate the associations between arthritis and the independent variables. Multivariate logistic regression was used to characterize the association between BMI and arthritis, and the final model consists of all covariates (sex, age, race/ethnicity, insurance status, and PA level) and 1 interaction (interaction between education and income), which was simultaneously entered into the model. Significance was established at *P* < .05. Data were weighted to adjust for differences in the probability of selection, nonresponse, and noncoverage of the survey, which often varies because of geographic location, availability of residential telephones, and number of adults per household. (For further discussion of data weighting, see http://www.cdc.gov/brfss/technicalinfodata/weighting.htm).

## Results

Women (51%) and men (49%) were almost equally represented, and 38% were aged 35 to 54 years (Table 1). Most were non-Hispanic white (69%) and had graduated from high school (87%). Most participants earned $50,000 or more and most had some type of insurance coverage (84%). Almost 60% of participants were in the overweight and obese range, 8% of whom were severely obese (BMI ≥35.0 kg/m^2^). Nearly half (48%) did not meet the recommended level of PA during the preceding month.

Among respondents who reported having arthritis, more women (59%) than men (41%) were affected. Participants in the oldest age category (≥65 years) comprised more than one-third of all cases, and more than 75% of reported arthritis was among non-Hispanic whites. Slightly more high school graduates (33%) reported arthritis compared with participants who had attended college (27%), and participants with less than a high school degree accounted for 14% of reported arthritis. The number of arthritis cases by income category did not vary greatly, and uninsured participants comprised only 10% of those reporting arthritis. Three-fourths of all arthritis cases were reported by people who engaged in some PA, and overweight and obese participants accounted for most cases (68%). Among participants at a normal weight, 40% reported no arthritis, and the proportion of participants without arthritis decreased dramatically as weight increased.

Overall, 26% of US adults reported doctor-diagnosed arthritis (Table 2). Higher prevalence of arthritis was associated with being female, older, and overweight or obese. Doctor-diagnosed arthritis was nearly twice as prevalent in obese participants (38%) compared with normal-weight participants (20%). Arthritis prevalence increased with increasing BMI category and with decreasing levels of PA. Compared with other participants, the prevalence of arthritis was higher for non-Hispanic whites, participants with low educational attainment, and participants in the lowest income category. Respondents who reported having insurance were more likely to report doctor-diagnosed arthritis (28%) than were respondents who reported not having insurance coverage (16%).

Age was the strongest predictor of arthritis in both the univariate and multivariate models (Table 3). Respondents in older age categories were significantly more likely to have arthritis, and participants aged 65 years or older were 13 times more likely to have arthritis (adjusted OR = 13.8; 95% CI, 13.0-14.7). The higher risk of arthritis among women remained stable after adjustment (unadjusted OR = 1.6; adjusted OR = 1.5). After adjustment for age, sex, race/ethnicity, socioeconomic variables, and levels of PA, high BMI remained a risk factor for arthritis. Participants in high BMI categories were significantly more likely to have arthritis compared with those in low BMI categories. Compared with normal-weight participants, the odds of arthritis were 3.3 times greater among participants who had class III obesity, 2.5 times greater among participants who had class II obesity, and 1.9 times greater among participants who had class I obesity. The odds of reporting arthritis increased by nearly 40% among participants who were overweight, and the association between BMI and arthritis remained after adjusting for covariates.

Respondents who reported having no insurance coverage were 52% less likely to report arthritis than those who reported having insurance coverage. After adjusting for all of the covariates, the association between uninsured status and arthritis weakened. Participants who reported having no coverage were only 20% less likely to report arthritis. The association between insufficient PA and arthritis was attenuated after adjustment (unadjusted OR = 1.2; adjusted OR = 1.0).

The interaction between education and income was significant (*P* = .047) (Table 3). Because education and income were not correlated (Pearson *r* = 0.0536), the final model consisted of all covariates and 1 interaction (interaction between education and income) (Table 4). Overall, risk of reporting arthritis was seen to decrease with increasing income category, regardless of educational attainment; the most pronounced decrease was observed among college graduates. Because education and income levels may vary by sex, we examined the interaction separately among men and women. The findings for men follow the trend observed for the overall results, while the findings for women were less consistent. The least educated women within the highest income category were at increased risk of reporting arthritis (OR = 1.1; 95% CI, 0.8-1.5).

## Discussion

### BMI and arthritis

The results of this study highlight the strong independent relationship between excess body weight and self-reported arthritis among adults in the United States. Because a more exclusive case definition of arthritis was used, we expected to find a lower risk of reported arthritis among all weight groups. However, our results were similar for people who were overweight and who had class I obesity, compared with a previous study, which used the old definition of arthritis, that reported similar ORs and 95% CIs for these weight categories (18). However, our results did differ among participants who had class II and class III obesity when compared with that same study, which found a lower odds of reporting arthritis among people with class II obesity (OR = 2.4; 95% CI, 2.1-2.5) and a higher odds of reporting arthritis among people with class III obesity (OR = 3.6; 95% CI, 3.2-3.8). The lower odds of reporting arthritis that we found for people with class III obesity may demonstrate that these people are more likely to report chronic joint symptoms than arthritis, but more research is needed in this area to be certain.

Our findings showed that the highest BMI category was associated with significantly higher odds of arthritis, and the association between high BMI and arthritis remained after adjusting for demographic and socioeconomic variables. Given the increasing prevalence of obesity in the United States and the alarming rate of increase among youth, arthritis symptoms may develop at a younger age. Clinicians should become aware of these trends and implement strategies to prevent obesity and reduce weight gain among this population. Physicians should recommend losing weight to their patients, and studies have shown that such counseling can promote weight-loss efforts among adults (19).

### Demographic and socioeconomic variables and arthritis

Age was the strongest independent predictor of arthritis, and this was not surprising given that previous studies have reported that nearly half of all arthritis cases occur in adults aged 65 years or older (20,21). The odds of reporting arthritis more than doubled from people aged 45 to 54 years to people aged 65 years or older, so it seems especially important for adults to maintain a healthy weight before they reach age 45.

After adjustment for all of the covariates included in the model, the risk of arthritis was 50% higher among women. Although the prevalence of obesity is higher among women than among men and although women typically live longer than men (22), our results show that the association between sex and arthritis is independent of BMI and age. Furthermore, the odds of women reporting arthritis remained high even after taking education and income into account. Biologic risk factors (eg, genetics, hormones) may explain the increased risk of arthritis among women (23,24), and more research is needed in these areas.

In unadjusted analyses, higher education and higher income categories were associated with significantly decreased odds of reporting arthritis. However, income and education were found to interact significantly, and results showed that the highest income earners, regardless of educational level, were less likely to report arthritis. These coincide with the results of previous research that indicated that the lowest income earners were at highest risk of arthritis (4). The association between low educational attainment and arthritis is less clear (25), and the interaction we detected may mask the true, independent association between income level and odds of having arthritis. Furthermore, the arthritis case definition we used required a diagnosis by a health professional. This fact may explain the decreased likelihood of participants without health insurance to report arthritis, because they may be more likely to not seek professional advice.

Non-Hispanic blacks were slightly less likely to report arthritis than were participants in the referent group, and Hispanics were nearly 50% less likely to report arthritis after adjustment. The prevalence of arthritis by race/ethnicity that we found was similar to that reported previously, with a higher prevalence of arthritis among whites (29%) and African Americans (26%) and a lower prevalence among Hispanics (15%) (20).

### Physical activity and arthritis

PA level was significantly associated with arthritis only among participants who did not participate in any type of PA during the preceding month. Participants who were inactive were 30% more likely to report arthritis, whereas the association between insufficient activity and arthritis was no longer significant after adjusting for all of the covariates and BMI. Our findings support the recommendations of the American College of Rheumatology for people to engage in recommended levels of PA to lower the risk of arthritis (26,27).

### Study limitations

Study findings are subject to certain limitations. Causal relationships cannot be inferred because the BRFSS is cross-sectional in design. Data obtained may be inaccurate because all variables rely on self-report; specifically, survey respondents often overreport height and underreport weight (28), and this fact may have resulted in lower estimates of BMI. Additionally, the case definition of arthritis we used excludes respondents who did not consult a health professional for their symptoms. Given that poor, uninsured people are more likely to be overweight and to forego formal medical treatment, results may not reflect the true association between BMI and arthritis (29). Furthermore, many types of arthritis vary significantly in etiology; some forms actually lead to weight gain (due to corticosteroid use and/or pain-related activity limitations). The BRFSS does not collect information on the type of arthritis or rheumatic disease or on the specific use of medications that could lead to weight gain, so study findings cannot be generalized to all people with arthritis. BRFSS is administered only to noninstitutionalized, nonmilitary adults, so findings may not be generalized to the entire US population.

### Conclusion

Arthritis and obesity are costly to the individual and the nation. As the US population ages and lives longer and as the prevalence of obesity continues to rise, arthritis may become an even greater public health concern. Achieving and maintaining a healthy weight may help delay the onset of arthritis. Given that body weight is modifiable, we have the opportunity to reduce the effects of arthritis. Our study results indicate that programs that target women who are overweight and members of the younger generation may have the greatest potential for decreasing arthritis prevalence among US adults. Future research is needed to determine whether maintaining a healthy weight delays the onset of arthritis and to investigate mechanisms by which excess body weight possibly leads to arthritis. More research will enhance efforts to address the public health challenges that arthritis and obesity create for our nation.

## Figures and Tables

**Table 1:** Respondent Characteristics (N = 356,112) by Arthritis Status, Behavioral Risk Factor Surveillance System, 2005

**Characteristic**	Total No. (%)	No. With Arthritis^a^ (%)	No. Without Arthritis (%)
**Total overall**	356,112 (100.0)	119,485 (26.0)	230,651 (71.8)
**Sex**			
Male	136,201 (48.5)	38,902 (40.6)	95,060 (51.4)
Female	219,911 (51.5)	80,583 (59.4)	135,591 (48.6)
**Age, y**			
18-34	64,903 (31.1)	5,690 (8.3)	58,184 (39.4)
35-44	63,425 (19.7)	11,414 (12.4)	51,003 (22.4)
45-54	73,297 (18.5)	23,195 (20.9)	49,005 (17.6)
55-64	64,441 (13.4)	29,774 (22.7)	33,719 (10.1)
≥65	87,351 (16.7)	48,673 (35.2)	36,986 (10.0)
**Race/Ethnicity**			
Non-Hispanic white	278,672 (68.7)	97,682 (76.4)	176,988 (66.5)
Non-Hispanic black	27,735 (9.4)	8,831 (9.3)	18,205 (9.4)
Hispanic	25,539 (14.8)	5,471 (8.3)	19,438 (16.8)
Other	20,750 (6.2)	6,277 (5.1)	14,044 (6.5)
**Education**			
Less than high school graduate	38,202 (12.5)	16,285 (13.9)	20,979 (11.7)
High school graduate	109,830 (29.9)	40,501 (32.6)	67,309 (28.9)
Attended college	93,228 (26.1)	31,682 (26.5)	60,264 (26.1)
College graduate	113,944 (31.3)	30,772 (26.7)	81,638 (33.2)
**Annual income, $**			
<25,000	94,577 (24.5)	40,499 (29.5)	52,477 (22.6)
25,000-49,999	94,113 (24.9)	30,749 (25.6)	62,257 (25.0)
≥50,000	118,676 (37.0)	30,343 (30.8)	87,065 (39.8)
**Insurance coverage**			
Insured	311,213 (83.6)	109,032 (90.1)	197,114 (81.4)
Uninsured	43,954 (16.0)	10,258 (9.7)	32,844 (18.1)
**Body mass index**			
Normal (<25.0 kg/m^2^)	129,513 (37.0)	34,853 (28.5)	92,653 (40.2)
Overweight (25.0-29.9 kg/m^2^)	123,692 (35.1)	41,718 (35.8)	80,168 (35.1)
Class I obesity (30.0-34.9 kg/m^2^)	55,599 (15.2)	22,761 (19.2)	32,098 (13.8)
Class II obesity (35.0-39.9 kg/m^2^)	19,439 (5.2)	9,025 (7.6)	10,176 (4.4)
Class III obesity (≥40.0 kg/m^2^)	11,419 (3.0)	5,976 (5.0)	5,291 (2.2)
**PA level^b^**			
Active	155,418 (44.7)	45,351 (39.3)	109,509 (47.8)
Insufficient activity	125,166 (35.0)	42,736 (35.9)	81,977 (35.6)
Inactive	50,399 (13.2)	23,502 (18.7)	26,582 (11.4)

Abbreviation: PA, physical activity.

a Respondents with arthritis were defined as those who answered yes to the question, "Have you ever been told by a doctor or other health professional that you have some form of arthritis, rheumatoid arthritis, gout, lupus, or fibromyalgia?"

b PA categories were based on the Surgeon General's recommendations (17). "Active" was defined as meeting the recommendations (30 minutes or more of moderate-intensity PA on 5 or more days per week or 20 minutes or more of vigorous-intensity PA on 3 or more days per week) during the preceding month, "insufficient activity" was defined as not meeting the recommendations during the preceding month, and "inactive" was defined as reporting no PA outside of one's occupation during the preceding month.

**Table 2:** Prevalence of Arthritis by Selected Characteristics, Behavioral Risk Factor Surveillance System, 2005

**Characteristic**	Arthritis Prevalence, % (95% CI)
**Total overall**	26.0 (26.0-26.0)
**Sex**	
Male	21.8 (21.8-21.8)
Female	30.1 (30.0-30.1)
**Age, y**	
18-34	7.0 (7.0-7.0)
35-44	16.4 (16.4-16.5)
45-54	29.4 (29.4-29.5)
55-64	44.1 (44.0-44.2)
≥65	54.8 (54.7-54.9)
**Race/Ethnicity**	
Non-Hispanic white	28.9 (28.9-29.0)
Non-Hispanic black	25.7 (25.6-25.8)
Hispanic	14.5 (14.5-14.6)
Other	21.3 (21.1-21.5)
**Education**	
Less than high school graduate	29.1 (29.0-29.3)
High school graduate	28.4 (28.3-28.5)
Attended college	26.5 (26.4-26.6)
College graduate	22.3 (22.2-22.4)
**Annual income, $**	
<25,000	31.4 (31.2-31.5)
25,000-49,999	26.7 (26.5-26.8)
≥50,000	21.7 (21.6-21.8)
**Insurance coverage**	
Insured	28.1 (28.0-28.2)
Uninsured	15.8 (15.7-16.0)
**Body mass index**	
Normal (<25.0 kg/m^2^)	20.1 (19.9-20.2)
Overweight (25.0-29.9 kg/m^2^)	26.5 (26.4-26.7)
Class I obesity (30.0-34.9 kg/m^2^)	33.0 (32.7-33.3)
Class II obesity (35.0-39.9 kg/m^2^)	37.9 (37.4-38.4)
Class III obesity (≥40.0 kg/m^2^)	44.0 (43.3-44.8)
**PA level^a^**	
Active	22.9 (22.7-23.1)
Insufficient activity	26.7 (26.5-26.9)
Inactive	37.1 (36.7-37.5)

Abbreviation: CI, confidence interval; PA, physical activity.

a PA categories were based on the Surgeon General's recommendations (17). "Active" was defined as meeting the recommendations (30 minutes or more of moderate-intensity PA on 5 or more days per week or 20 minutes or more of vigorous-intensity PA on 3 or more days per week) during the preceding month, "insufficient activity" was defined as not meeting the recommendations during the preceding month, and "inactive" was defined as reporting no PA outside of one's occupation during the preceding month..

**Table 3:** Unadjusted and Adjusted Risk of Arthritis, by Covariates, Behavioral Risk Factor Surveillance System, 2005^a^

**Characteristic**	Unadjusted OR (95% CI)	Adjusted OR (95% CI)
**Sex**		
Male	1 [Reference]	1 [Reference]
Female	1.6 (1.5-1.6)	1.5 (1.5-1.6)
**Age, y**		
18-34	1 [Reference]	1 [Reference]
35-44	2.6 (2.5-2.8)	2.5 (2.4-2.7)
45-54	5.6 (5.3-5.9)	5.2 (4.9-5.5)
55-64	10.6 (10.0-11.3)	9.5 (8.9-10.0)
≥65	16.7 (15.7-17.6)	13.8 (13.0-14.7)
**Race/Ethnicity**		
Non-Hispanic white	1 [Reference]	1 [Reference]
Non-Hispanic black	0.9 (0.8-0.9)	0.8 (0.8-0.9)
Hispanic	0.4 (0.4-0.5)	0.5 (0.5-0.6)
Other	0.7 (0.6-0.7)	0.9 (0.8-1.0)
**Insurance coverage**		
Insured	1 [Reference]	1 [Reference]
Uninsured	0.5 (0.5-0.5)	0.8 (0.7-0.8)
**Body mass index**		
Normal (<25.0 kg/m2)	1 [Reference]	1 [Reference]
Overweight (25.0-29.9 kg/m^2^)	1.4 (1.4-1.5)	1.4 (1.3-1.4)
Class I obesity (30.0-34.9 kg/m^2^)	2.0 (1.9-2.1)	1.9 (1.8-2.0)
Class II obesity (35.0-39.9 kg/m^2^)	2.4 (2.3-2.6)	2.5 (2.3-2.7)
Class III obesity (≥40.0 kg/m^2^)	3.1 (2.9-3.4)	3.3 (3.1-3.6)
**PA level^b^**		
Active	1 [Reference]	1 [Reference]
Insufficient activity	1.2 (1.2-1.3)	1.0 (1.0-1.1)
Inactive	2.0 (1.9-2.1)	1.3 (1.2-1.3)

Abbreviations: OR, odds ratio; CI, confidence interval; PA, physical activity.

a Because the education and income variables were not correlated (Pearson *r* = 0.0536), the final model used to derive the adjusted values consisted of all independent covariates and the interaction between education and income; all predictors were simultaneously entered into the model (*P* = .047).

b PA categories were based on the Surgeon General's recommendations (17). "Active" was defined as meeting the recommendations (30 minutes or more of moderate-intensity PA on 5 or more days per week or 20 minutes or more of vigorous-intensity PA on 3 or more days per week) during the preceding month, "insufficient activity" was defined as not meeting the recommendations during the preceding month, and "inactive" was defined as reporting no PA outside of one's occupation during the preceding month.

**Table 4:** Interaction Between Education and Annual Income and Risk of Arthritis, Overall and Stratified by Sex, Behavioral Risk Factor Surveillance System, 2005

**Education**	Income, $	Overall OR (95% CI **)**	Men OR (95% CI **)**	Women OR (95% CI)
Less than high school graduate	<25,000	1.0 [Reference]	1.0 [Reference]	1.0 [Reference]
Less than high school graduate	25,000-49,999	0.9 (0.7-1.0)	0.9 (0.7-1.1)	0.8 (0.7-1.0)
Less than high school graduate	≥50,000	0.8 (0.7-1.0)	0.6 (0.5-0.9)	1.1 (0.8-1.5)
High school graduate	<25,000	0.9 (0.9-1.0)	0.9 (0.8-1.0)	0.9 (0.9-1.0)
High school graduate	25,000-49,999	0.7 (0.6-0.9)	0.7 (0.5-1.0)	0.7 (0.5-1.0)
High school graduate	≥50,000	0.6 (0.5-0.8)	0.6 (0.4-0.9)	0.7 (0.5-0.9)
Attended college	<25,000	1.0 (0.9-1.1)	0.9 (0.8, 1.1)	1.0 (0.9-1.2)
Attended college	25,000-49,999	0.8 (0.6-1.0)	0.7 (0.5-1.0)	0.8 (0.5-1.1)
Attended college	≥50,000	0.6 (0.5-0.8)	0.6 (0.4-0.9)	0.6 (0.4-0.8)
College graduate	<25,000	0.8 (0.7-0.9)	0.7 (0.6-0.9)	0.9 (0.7-1.0)
College graduate	25,000-49,999	0.6 (0.5-0.8)	0.6 (0.4-0.8)	0.7 (0.5-0.9)
College graduate	≥50,000	0.5 (0.4-0.6)	0.5 (0.3-0.7)	0.5 (0.4-0.7)

Abbreviations: OR, odds ratio; CI, confidence interval.
